# Bufalin targets E2F2 to transcriptionally inhibit LINC01410 and suppress Wnt/β-catenin signaling in esophageal squamous cell carcinoma

**DOI:** 10.1038/s41698-026-01395-0

**Published:** 2026-05-18

**Authors:** Xiaohong Kang, Yufei Ma, Hongke Zhao, Haoling Zhang, Mridul Roy, Mingjuan Liao, Ying Wei, Xingqiao Li, Yueyang Zhou, Huange Xue, Xiaoxia Yan, Wangzheqi Zhang, Yadong Guo, Fei Cao, Tingmin Chang

**Affiliations:** 1 Department of Oncology, The first Affiliated Hospital of Henan Medical University, Xingxiang, Henan China; 2 Life Science Research Center, The first Affiliated Hospital of Henan Medical University, Xinxiang, Henan China; 3 Department of Traditional Chinese Medicine, Shanghai Ninth People’s Hospital of Shanghai Jiao Tong University, Shanghai, China; 4 Department of Oncology, Luoyang Orthopedic-traumatological Hospital of Henan province, Luoyang, Henan China; 5 School of Anesthesiology, Naval Medical University, Shanghai, China; 6 Department of Urology, Shanghai Tenth People’s Hospital, Shanghai, China; 7 Department of Gastroenterology, The first Affiliated Hospital of Henan Medical University, Xinxiang, Henan China

**Keywords:** Cancer, Cell biology, Molecular biology, Oncology

## Abstract

Bufalin, a main active monomer component extracted from the Traditional Chinese Medicine toad venom, exhibits potent anti-tumour activity across diverse malignancies. However, its specific molecular targets in Oesophageal squamous cell carcinoma (ESCC) remain unclear. Here, we demonstrate that bufalin suppresses ESCC growth and metastasis in a concentration-dependent manner both in vitro and in vivo. RNA sequencing analysis revealed that bufalin inhibits ESCC progression by downregulating LINC01410. TCGA-ESCC data indicated that LINC01410 is highly expressed in ESCC tissues and promotes tumour progression by stabilising β-catenin protein and activating the Wnt/β-catenin pathway. Through multiple approaches including proteome microarray screening and machine learning analyses, we identified E2F2 as a upstream transcription factor of LINC01410. We further demonstrate that bufalin induces the proteasomal degradation of E2F2, consequently silencing LINC01410 transcription and collapsing the LINC01410-Wnt/β-catenin signalling axis. In summary, our findings indicate that bufalin targets E2F2 to downregulate LINC01410, thereby providing a potential therapeutic strategy for suppressing ESCC progression.

## Introduction

Esophageal cancer is the most common malignant tumour of the digestive system and ranks seventh in global cancer mortality^1^. Histologically, esophageal squamous cell carcinoma (ESCC) predominates in East Asian populations, comprising 85% of cases^2,3^. Asymptomatic onset frequently delays diagnosis until advanced stages. Despite multimodal therapy, including chemotherapy, radiotherapy, and immunotherapy, the 5-year overall survival rate of patients with metastatic ESCC remains approximately 5%^4,5^. Identifying effective therapeutic targets to develop improved anti-cancer drugs is therefore a clinical priority.

The Wnt/β-catenin signalling pathway, as a key pathway regulating cell development, differentiation, and proliferation, plays a central role in the occurrence and progression of ESCC. Its abnormal activation can promote tumour cell proliferation, epithelial-mesenchymal transition (EMT) process, and distant metastasis, which is closely associated with the poor prognosis of ESCC patients, making it a highly promising therapeutic target for ESCC^6^. Long non-coding RNAs (lncRNAs) are defined as non-coding RNAs (ncRNAs) exceeding 200 nucleotides with limited protein-coding capacity^7^. Increasing evidence shows that lncRNAs are dysregulated in various malignant tumours and can participate in tumour progression by regulating key signalling pathways such as Wnt/β-catenin, thereby modulating critical biological behaviours of tumour cells including proliferation, apoptosis, invasion, and metastasis^8–13^. Long intergenic non-coding RNA 01410 (LINC01410) represents a novel lncRNA highly expressed in diverse cancers, driving progression in malignancies such as bladder cancer and osteosarcoma^14,15^. Our previous studies and independent reports indicate that in ESCC, high LINC01410 expression accelerates EMT and promotes proliferation and metastasis^16^, suggesting LINC01410 is a viable therapeutic target. Yet, the upstream drivers of LINC01410 upregulation and the precise mechanisms governing its promotion of ESCC progression remain undefined, necessitating the development of specific inhibitors.

Natural products support the discovery of anti-cancer drugs^17^, and identifying their specific targets is essential for innovative therapy development. Bufalin is an active monomer extracted from *Venenum bufonis* clinically utilised in China to treat various cancers^18^. Studies indicate that bufalin exerts effective anti-tumour effects against lung, liver, colorectal, and prostate cancers^19–21^. Pharmacological studies confirm that bufalin inhibits multiple signalling pathways, including Hippo-YAP, Wnt/β-catenin, JNK, SRC3/MIF, mTOR, and MAPK, inducing apoptosis, necrosis, and autophagy in cancer cells^22,23^. However, the specific molecular mechanisms and targets of bufalin in ESCC remain unclear.

Here, we applied RNA sequencing to investigate the target genes of bufalin against ESCC and observed that bufalin inhibits ESCC cell proliferation, invasion, and metastasis by downregulating LINC01410. Then, we observed that LINC01410 binds to β-catenin in ESCC, activating the Wnt/β-catenin pathway by increasing β-catenin protein stability. Proteomic microarray screening and machine learning approaches identified E2F2 as a transcription factor of LINC01410; bufalin targets and degrades E2F2, inhibiting the LINC01410-Wnt/β-catenin signalling axis and preventing ESCC progression. This study suggests that bufalin, by targeting and inhibiting E2F2/LINC01410 in ESCC, as a potetial therapeutic candidate for ESCC.

## Results

### Bufalin inhibits ESCC tumour growth and metastasis both in vitro and in vivo

**Fig. 1:**
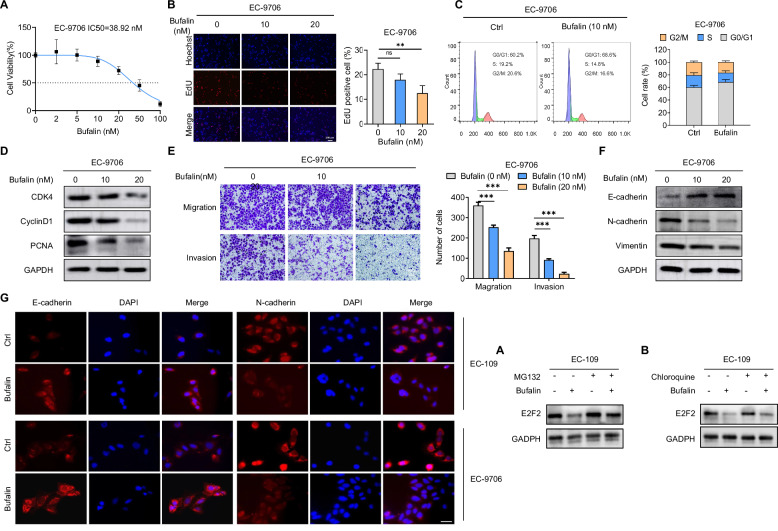
Bufalin inhibits the tumorigenesis and metastasis of ESCC in vitro and in vivo. **A** Cell viability of EC-109 cells treated with different concentrations of bufalin for 48 h, detected by CCK-8 assay; **B** EdU (red, marker for proliferating cells) and Hoechst (blue, marker for cell nuclei) staining results of ESCC cells after bufalin treatment; **C** Cell cycle distribution of ESCC cells after bufalin treatment, analyzed by flow cytometry; **D** Protein levels of Cyclin D1, CDK4, and PCNA in EC-109 cells after bufalin treatment, detected by Western blot (GAPDH used as internal control); **E** Migration and invasion abilities of EC-109 cells after bufalin treatment, detected by Transwell assay; **F** Protein expression levels of E-cadherin, N-cadherin, and Vimentin analyzed by Western blot; **G**, **H** Photographs of tumour tissues obtained from tumour-bearing mice sacrificed at the end of drug administration; **I** Growth curves of EC-109 xenografts in the control group and bufalin-treated group; **J** Statistical analysis of tumour weights at the end of the experiment; **K** Immunohistochemical (IHC) staining and quantitative analysis of Ki-67 in xenografts; **L** H&E staining results of mouse lung tissues.

To evaluate the anti-tumour efficacy of bufalin in ESCC, we assessed ESCC cell proliferation. The results showed that bufalin significantly inhibited the proliferation of EC-9706 and EC-109 cells in a concentration-dependent manner, with concurrent downregulation of the proliferation marker PCNA (Fig. 1A, B and Supplementary Fig. S1A, B, Fig. 1D and Supplementary Fig. S1D). Further studies revealed that this inhibition was associated with cell cycle regulation; bufalin significantly induced G0/G1 phase arrest in both EC-9706 and EC-109 cell lines (Fig. 1C and Supplementary Fig. S1C) and downregulated the G1 checkpoint regulators Cyclin D1 and CDK4 (Fig. 1D and Supplementary Fig. S1D).

Transwell assays demonstrated that bufalin treatment (24 h) dose-dependently reduced migration and invasion capabilities (Fig. 1E and Supplementary Fig. S1E). Concomitantly, the epithelial marker E-cadherin was upregulated, whereas mesenchymal markers N-cadherin and Vimentin were downregulated, indicating that bufalin impairs ESCC motility by suppressing epithelial-mesenchymal transition (EMT) (Fig. 1F and Supplementary Fig. S1F, G)^24^. These results reveal that bufalin restricts ESCC tumorigenicity in vitro.

To validate these findings in vivo, we established an EC-109 subcutaneous xenograft mouse model (Fig. 1G). In vivo experiments showed that bufalin treatment significantly inhibited subcutaneous tumour growth (Fig. 1H, I) and reduced tumour weight (Fig. 1J). Immunohistochemical analysis showed that the proliferation marker Ki-67 was significantly downregulated in treated tumour tissues (Fig. 1K). In the lung metastasis model, bufalin effectively inhibited distant dissemination, significantly reducing the number of metastatic nodules (Fig. 1L). These data demonstrate the capability of bufalin to suppress ESCC tumour growth and metastasis.

### Bufalin downregulates the expression of LINC01410 in ESCC

**Fig. 2:**
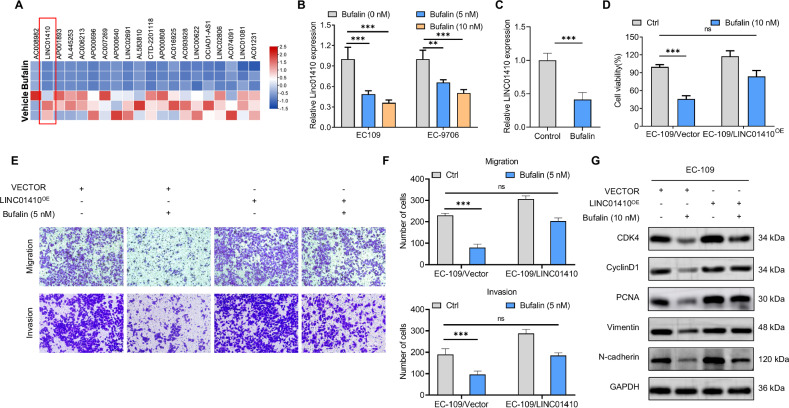
Bufalin downregulates LINC01410 expression in ESCC. **A** Heatmap of RNA-seq data showing differentially expressed lncRNAs in EC-109 cells after bufalin treatment (red indicates high expression, blue indicates low expression); **B** RNA expression levels of LINC01410 in EC-109 and EC-9706 cells, verified by qRT-PCR; **C** Quantitative analysis of LINC01410 RNA expression in mouse tumour tissues; **D** CCK-8 assay showing that LINC01410 overexpression reverses bufalin-induced inhibition of cell proliferation; **E**, **F** Migration and invasion abilities of LINC01410-overexpressing ESCC cells treated with bufalin, detected by Transwell assay; cell numbers per field were quantified using ImageJ; **G** Protein levels of Cyclin D1, CDK4, PCNA, N-cadherin, and Vimentin in LINC01410-overexpressing cells after bufalin treatment, detected by Western blot (GAPDH used as internal control).

Bufalin downregulates LINC01410 expression in ESCC Dysregulation of lncRNA expression plays a key role in ESCC progression^25^. To investigate the effect of bufalin on lncRNA expression, we performed RNA sequencing on bufalin-treated EC-109 cells. Results showed that 62 lncRNAs were significantly downregulated ( | fold change | ≤0.5, *P* < 0.01), with LINC01410 showing the most significant decrease (Fig. 2A and Supplementary Table S2). Given that LINC01410 is overexpressed in various cancers and promotes EMT and metastasis in ESCC^14,15^, we hypothesised that it is a key target of bufalin.

Validation experiments showed that bufalin treatment significantly downregulated LINC01410 mRNA levels in EC-109 and EC-9706 cells (Fig. 2B). Similarly, in the EC-109 xenograft model, bufalin administration reduced LINC01410 mRNA levels in tumour tissue (Fig. 2C). Functional rescue experiments showed that LINC01410 overexpression significantly reversed bufalin-mediated growth inhibition (Fig. 2D). Transwell assays further showed that LINC01410 overexpression attenuated the inhibitory effects of bufalin on migration and invasion (Fig. 2E, F). Western blot analysis demonstrated that LINC01410 overexpression weakened the bufalin-induced downregulation of Cyclin D1, CDK4, PCNA, N-cadherin, and Vimentin (Fig. 2G). Together, these findings suggest that the anti-tumour effects of bufalin in ESCC depend on the downregulation of LINC01410.

### LINC01410 promotes Wnt/β-catenin signalling in ESCC

**Fig. 3:**
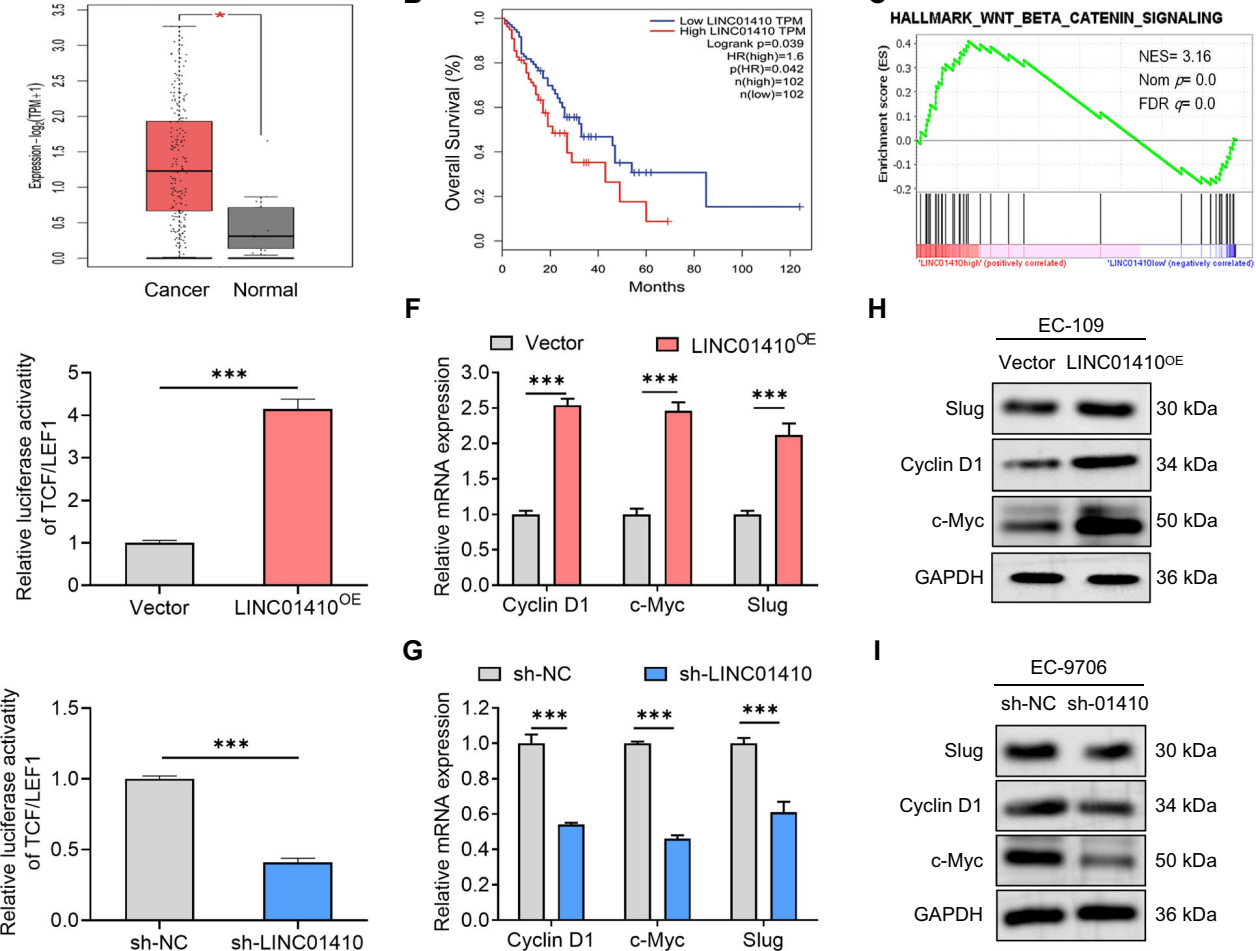
LINC01410 activates the Wnt/β-catenin signalling in ESCC. **A** Expression difference of LINC01410 between ESCC tumour tissues and normal tissues, analyzed using TCGA dataset; **B** Kaplan–Meier survival analysis of ESCC patients stratified by LINC01410 expression level; **C** GSEA analysis showing significant enrichment of the Wnt/β-catenin pathway in the high-LINC01410 expression group (NES = 3.16, nom *p* = 0.0, FDR *q* = 0.0); **D**, **E** Wnt signalling activity in EC-109 cells with LINC01410 overexpression or EC-9706 cells with LINC01410 knockdown, detected by TCF/LEF luciferase reporter assay; **F**, **G** mRNA expression levels of Wnt target genes (Cyclin D1, c-Myc, Slug) in cells with LINC01410 overexpression or knockdown, analyzed by qRT-PCR; **H**, **I** Protein levels of Wnt target genes in cells with LINC01410 overexpression or knockdown, detected by Western blot (GAPDH used as internal control).

To further explore the role of LINC01410 in ESCC tumour growth, we analysed The Cancer Genome Atlas (TCGA) data via GEPIA2. LINC01410 expression was significantly higher in ESCC tumour samples than in normal tissues (Fig. 3A). Kaplan–Meier analysis showed that high LINC01410 expression correlates with shorter overall survival (Fig. 3B), suggesting that LINC01410 may be a poor prognostic marker for ESCC. To identify potential signalling pathways, we used KEGG and Gene Set Enrichment Analysis (GSEA). Results showed that the Wnt/β-catenin pathway was significantly enriched in patients with high LINC01410 expression (Fig. 3C and Supplementary Fig. S2).

To determine the relationship between LINC01410 and the Wnt/β-catenin pathway, we treated ESCC cells with the Wnt inhibitor XAV939 (20 μM). XAV939 treatment did not significantly affect LINC01410 expression (data not shown). Conversely, TCF/LEF1 luciferase reporter assays showed that LINC01410 knockdown significantly reduced, while overexpression enhanced, Wnt signalling activity (Fig. 3D, E). qRT-PCR results showed that LINC01410 overexpression upregulated the mRNA levels of Wnt target genes (Cyclin D1, c-Myc, and Slug) in EC-109 cells (Fig. 3F), while knockdown reduced their expression (Fig. 3G). Western blotting supported these findings: LINC01410 overexpression in EC-109 cells increased, whereas its knockdown in EC-9706 cells decreased, the protein levels of these Wnt/β-catenin target genes (Fig. 3H, I). These results indicate that LINC01410 positively regulates the Wnt/β-catenin signalling pathway in ESCC.

### LINC01410 enhances Wnt/β-catenin signalling in ESCC through stabilisation of β-catenin protein

**Fig. 4:**
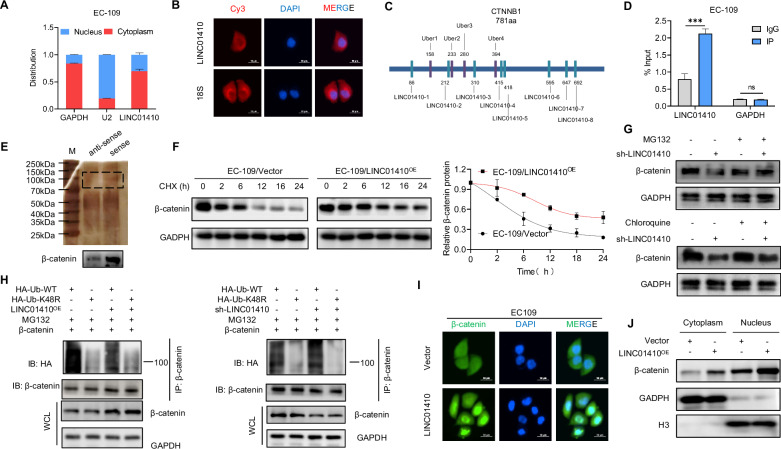
LINC01410 activates Wnt/β-catenin signalling by stabilising the β-catenin protein in ESCC. **A** Subcellular distribution of LINC01410 in ESCC cells, analyzed by RNA subcellular fractionation assay; **B** Cellular localisation of LINC01410 detected by RNA FISH (red: LINC01410; blue: cell nuclei); **C** Prediction of the interaction between LINC01410 and β-catenin using RNAInter, GPSv6.0, and GPS-Uber databases; **D** Verification of the interaction between LINC01410 and β-catenin by RIP assay; **E** Silver staining of RNA pull-down products after PAGE electrophoresis, followed by Western blot detection of β-catenin; **F** Stability of β-catenin protein in LINC01410-overexpressing cells, evaluated by CHX chase assay; **G** Expression of β-catenin in LINC01410-knockdown cells co-incubated with the proteasome inhibitor MG132 or the lysosome inhibitor chloroquine, analyzed by Western blot; **H** Ubiquitination level of β-catenin in 293 T cells with LINC01410 knockdown or overexpression, detected by co-immunoprecipitation assay; **I** Immunofluorescence staining of β-catenin in LINC01410-overexpressing cells (green: β-catenin; blue: cell nuclei); **J** Distribution of β-catenin in the cytoplasm and nucleus of EC-109 cells, analyzed by Western blot (GAPDH used as cytoplasmic internal control; Histone H3 used as nuclear internal control).

Inhibition of GSK-3β reduces β-catenin phosphorylation, stabilising the protein and promoting nuclear translocation to regulate gene expression. Based on this, we hypothesised that LINC01410 might activate Wnt/β-catenin signalling by regulating β-catenin stability. Subcellular fractionation and RNA fluorescence in situ hybridisation (FISH) showed that LINC01410 is predominantly cytoplasmic (Fig. 4A, B), providing the spatial basis for its interaction with β-catenin. RNAInterv4.0 analysis predicted β-catenin as a potential binding partner of LINC01410, while GPSv6.0 and GPS-Uber predicted that LINC01410 binding might protect β-catenin from degradation (Fig. 4C). To validate this interaction, we performed RNA immunoprecipitation (RIP) and RNA pull-down assays. RIP results showed significant enrichment of LINC01410 in β-catenin complexes (Fig. 4D). Furthermore, incubation of cell lysates with in vitro synthesised LINC01410 (sense and antisense) followed by silver staining and Western blotting revealed a specific 90 kDa band corresponding to β-catenin bound to LINC01410 (Fig. 4E). FISH analysis further showed cytoplasmic co-localisation of LINC01410 and β-catenin (Supplementary Fig. S3), confirming their direct interaction.

To determine the effect of LINC01410 on β-catenin stability, we treated cells with the protein synthesis inhibitor cycloheximide (CHX). Results showed that LINC01410 overexpression extended the half-life of β-catenin in EC-109 cells (Fig. 4F). Treatment with the proteasome inhibitor MG132 reversed the β-catenin depletion caused by LINC01410 knockdown (Fig. 4G), suggesting that LINC01410 stabilises β-catenin by inhibiting the proteasome pathway. Immunoprecipitation assays showed that LINC01410 regulates K48-linked ubiquitination of β-catenin. Overexpression significantly reduced, while knockdown increased, β-catenin polyubiquitination levels (Fig. 4H). Finally, immunofluorescence and Western blot experiments showed that LINC01410 overexpression promoted β-catenin nuclear translocation (Fig. 4I, J). Together, these data suggest that LINC01410 binds directly to β-catenin, inhibits its K48-mediated ubiquitination to enhance stability, and promotes nuclear translocation, thereby activating Wnt/β-catenin signalling.

### Bufalin inhibits ESCC cell growth and migration by suppressing LINC01410-mediated Wnt/β-catenin signalling

**Fig. 5:**
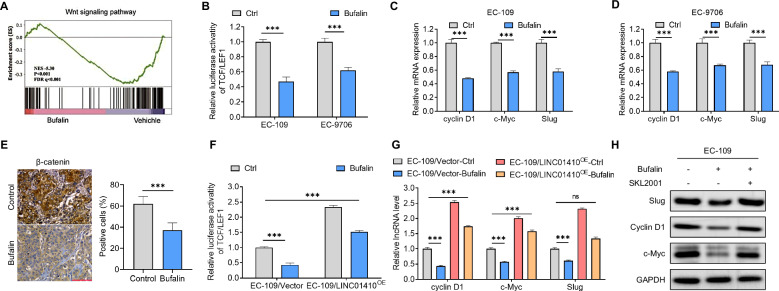
Bufalin attenuates ESCC cell growth and migration by inhibiting LINC01410-mediated Wnt/β-catenin signalling. **A** GSEA analysis of RNA-seq data from EC-109 cells treated with bufalin (enrichment in the Wnt/β-catenin pathway); **B** TCF/LEF luciferase reporter activity in cells after bufalin treatment; **C**, **D** mRNA expression levels of Wnt target genes (Cyclin D1, c-Myc, Slug) in cells after bufalin treatment, analyzed by qRT-PCR; **E** IHC staining and quantitative analysis of β-catenin in xenografts; **F** TCF/LEF reporter activity in LINC01410-overexpressing cells treated with bufalin; **G** mRNA expression levels of Wnt target genes in LINC01410-overexpressing or knockdown cells treated with bufalin, analyzed by qRT-PCR; **H** Protein levels of Wnt targets in cells co-treated with SKL2001 and bufalin, detected by Western blot (GAPDH used as internal control).

To identify the signalling pathways underlying the anti-ESCC effects of bufalin, we performed RNA sequencing (RNA-seq) on bufalin-treated EC-109 cells. Gene Set Enrichment Analysis (GSEA) showed that differentially expressed genes were significantly enriched in the Wnt signalling pathway (Fig. 5A). Given that LINC01410 activates Wnt/β-catenin signalling, we hypothesised that bufalin might suppress ESCC growth and migration by suppressing the LINC01410-mediated Wnt/β-catenin signalling axis. TCF/LEF1 luciferase reporter assays showed that bufalin treatment significantly reduced Wnt signalling activity (Fig. 5B) and significantly downregulated the mRNA levels of Wnt target genes (Cyclin D1, c-Myc, Slug) in ESCC cells (Fig. 5C, D). Immunohistochemistry (IHC) further showed that bufalin significantly reduced β-catenin expression in mouse xenograft tumours (Fig. 5E).

To determine whether bufalin mediated Wnt inhibition depends on LINC01410, we treated LINC01410-overexpressing EC-109 cells with bufalin. Luciferase assays showed that LINC01410 overexpression significantly attenuated bufalin-induced Wnt inhibition (Fig. 5F). qRT-PCR results showed that LINC01410 overexpression reversed the bufalin-mediated downregulation of Wnt targets (Cyclin D1, c-Myc, Slug) (Fig. 5G). Moreover, the Wnt pathway agonist SKL2001 counteracted the effect of bufalin on Wnt target expression (Fig. 5H). Together, these findings suggest that bufalin inhibits ESCC growth and migration by suppressing the LINC01410-mediated Wnt/β-catenin signalling pathway.

### Bufalin inhibits LINC01410 expression by inducing E2F2 degradation

**Fig. 6:**
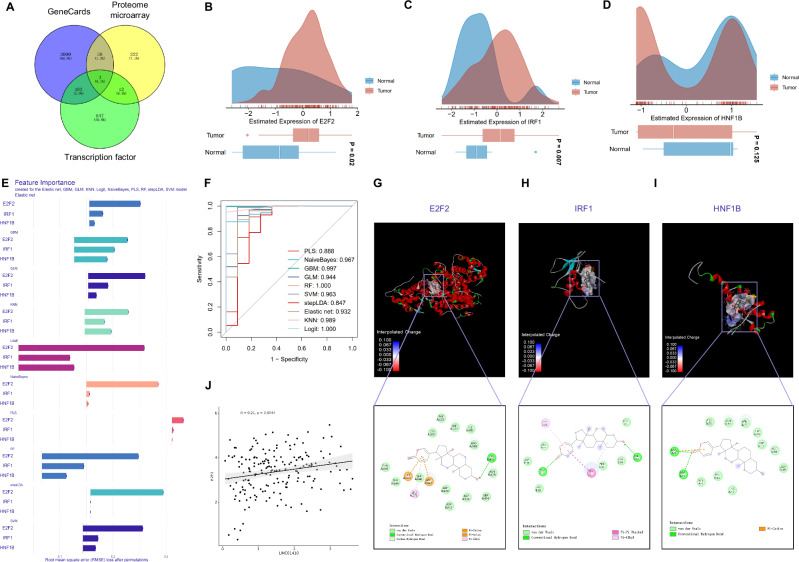
Prediction of Bufalin target proteins in esophageal carcinoma. **A** Venn diagram showing the intersection of proteins identified by proteome microarray, disease targets from the GeneCards database, and transcription factors; **B**–**D** Expression analysis of E2F2, IRF1, and HNF1B genes in the TCGA-ESCA cohort; **E** Ranking of the top 10 most important genes selected by 10 machine learning models (ElasticNet, GBM, GLM, KNN, Logit, NaiveBayes, PLS, RF, stepLDA, and SVM) based on permutation root mean square error (RMSE) loss; **F** ROC curves and AUC values of the 10 machine learning models; **G**–**I** Molecular docking analysis of bufalin (PubChem CID: 9547215) with E2F2 (PDB: 1N4M), IRF1 (PDB: 1IF1), or HNF1B (PDB: 2DA6) using PyRx software; (**J**) Correlation scatter plot of E2F2 and LINC01410 expression.

To elucidate the mechanism underlying LINC01410 suppression, we examined whether bufalin targets upstream transcriptional regulators. We integrated 275 potential bufalin-binding proteins identified by proteomic microarray screening^19,26–28^, 2,233 disease targets from GeneCards, and 875 transcription factors. Intersection analysis identified E2F2, IRF1, and HNF1B as potential bufalin-associated transcription factors (Fig. 6A). TCGA-ESCA cohort analysis showed that E2F2 and IRF1 were significantly overexpressed in ESCC tissues (Fig. 6B–D). To prioritise the key regulator, we systematically compared the predictive performance of these targets using ten machine learning models (including RF, GBM, SVM, and KNN). Feature importance analysis showed that HNF1B, IRF1, and E2F2 exhibited high consistency across all models (Fig. 6E). Residual box plots and inverse cumulative distribution results indicated that RF, GBM, SVM, and KNN models achieved the highest fitting accuracy and stability (Supplementary Fig. S4). ROC curve analysis confirmed that the GBM model performed best (AUC = 0.997), with KNN and SVM also showing excellent classification capabilities (Fig. 6F). Based on these metrics, E2F2 was identified as the most discriminative candidate.

Molecular docking studies indicated that E2F2 had the strongest binding affinity to bufalin, and E2F2 expression was significantly and positively correlated with LINC01410 expression (Fig. 6G-J and Supplementary Table S1). Notably, E2F2 and LINC01410 levels were significantly elevated in esophageal cancer tissues compared to normal tissues; this upregulation was observed from Stage 1 and persisted throughout all subsequent stages (Supplementary Fig. S5). This suggests that dysregulation of the E2F2/LINC01410 axis is an early event in ESCC progression.

**Fig. 7:**
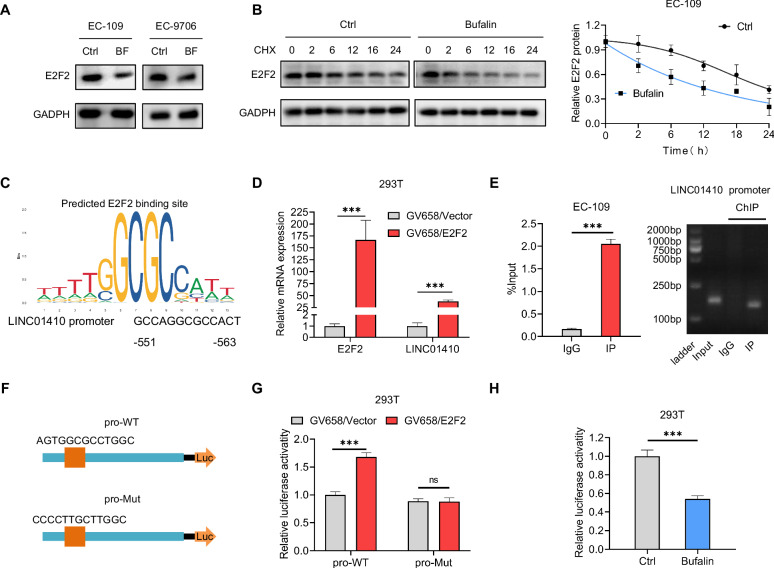
Bufalin inhibits LINC01410 expression by degrading E2F2. **A** Protein level of E2F2 in cells treated with bufalin, detected by Western blot (GAPDH used as internal control); **B** Stability of E2F2 evaluated by CHX chase assay (Ctrl control group, BF bufalin-treated group); **C** Binding sites of E2F2 on the LINC01410 promoter, predicted by the JASPAR database; **D** Expression level of LINC01410 in E2F2-overexpressing cells, analyzed by qRT-PCR; **E** Verification of the binding between E2F2 and the LINC01410 promoter by ChIP-qPCR; **F** Schematic of wild-type (pro-WT) and mutant (pro-Mut) LINC01410 promoter constructs used in the dual-luciferase assay; **G** Comparison of luciferase activity between wild-type and mutant LINC01410 promoters in E2F2-overexpressing cells; **H** Luciferase activity of the LINC01410 promoter in cells after bufalin treatment.

As aberrant E2F2 expression is associated with poor prognosis in various cancers^29^, we hypothesised that bufalin inhibits LINC01410 transcription by reducing E2F2 protein levels. Western blot analysis showed that E2F2 protein levels were significantly lower in bufalin-treated cells compared to untreated controls (Fig. 7A). Cycloheximide (CHX) chase assays showed that bufalin treatment accelerated the degradation rate of E2F2 (Fig. 7B). Furthermore, the proteasome inhibitor MG132 reversed bufalin-induced E2F2 depletion (Supplementary Fig. S6), indicating that bufalin promotes E2F2 degradation via the proteasome pathway.

To determine if E2F2 regulates LINC01410 transcription, we consulted the JASPAR database, which predicted E2F2 binding sites within the LINC01410 promoter (Fig. 7C). Consistent with this, E2F2 overexpression increased LINC01410 levels (Fig. 7D). Chromatin immunoprecipitation (ChIP) assays confirmed that E2F2 binds directly to the LINC01410 promoter (Fig. 7E). To validate transcriptional regulation, we constructed wild-type (pro-WT) and mutant (pro-Mut) LINC01410 promoter vectors (Fig. 7F). Luciferase assays showed that E2F2 positively regulates LINC01410 promoter activity (Fig. 7G). Finally, dual-luciferase assays showed that bufalin significantly inhibited LINC01410 promoter activity (Fig. 7H). Together, these results suggest that bufalin inhibits LINC01410 transcription by inducing E2F2 degradation.

## Discussion

**Fig. 8:**
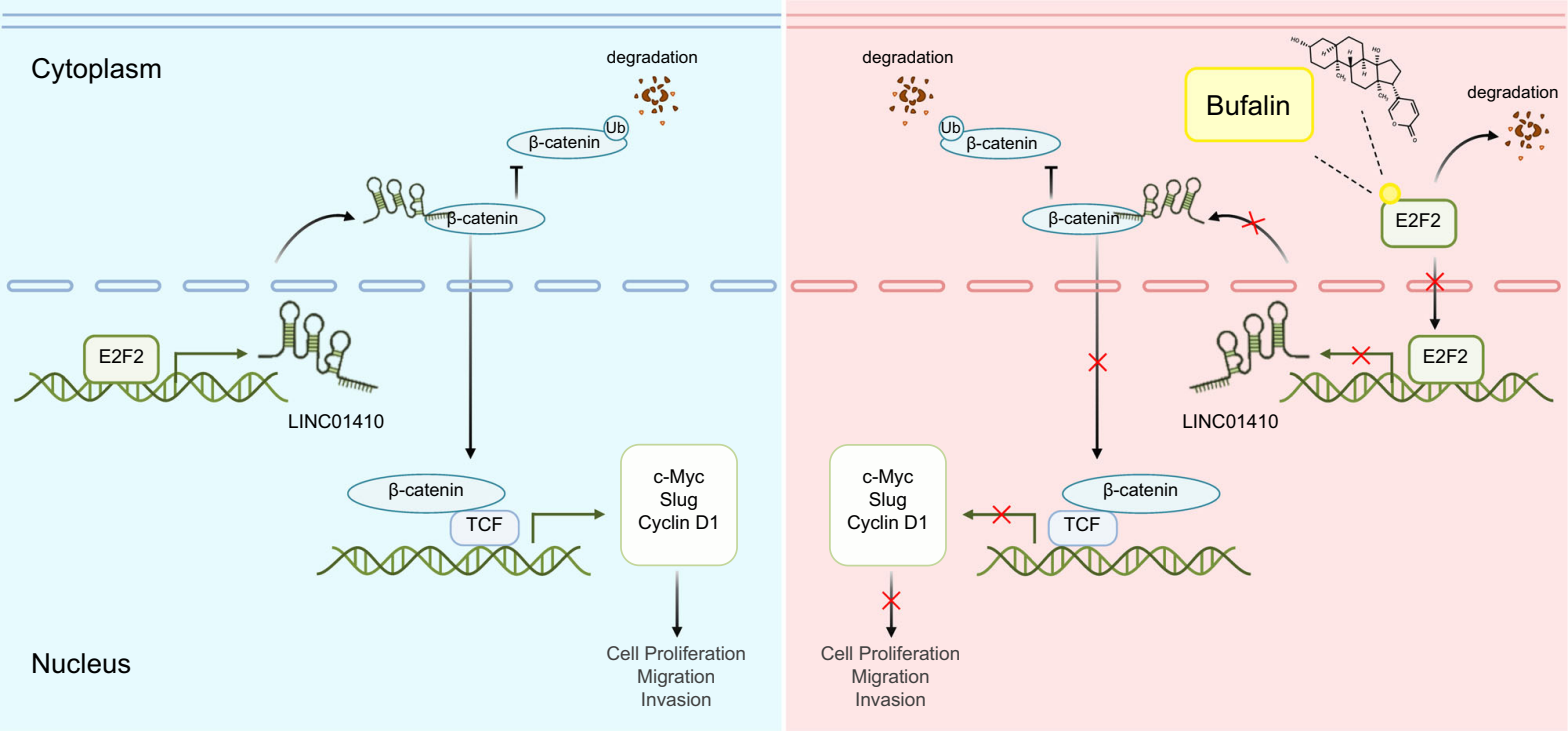
Schematic representation of the proposed mechanism underlying the antitumor activity of bufalin in ESCC. LINC01410 plays a crucial role in the Wnt/β-catenin signalling pathway by stabilising β-catenin, which in turn activates the pathway, promoting cell growth and epithelial-mesenchymal transition (EMT) in ESCC cells. Bufalin is suggested to downregulate LINC01410 by inducing the degradation of E2F2, a transcription factor that binds to the LINC01410 promoter, thereby leading to the inhibition of ESCC cell growth, EMT, and metastasis by disrupting the E2F2/LINC01410/Wnt/β-cateni signalling axis.

Bufalin is a clinically validated natural product with promising therapeutic potential across diverse tumour types. Pharmacological studies have revealed that Bufalin exerts its anti-tumour effects by promoting cell senescence, arresting the cell cycle, inhibiting cell proliferation, preventing angiogenesis, and inducing apoptosis^30–32^. However, its molecular targets in ESCC remain poorly defined. Although dysregulation of lncRNAs is increasingly recognised as a driver of tumour progression, the impact of bufalin on the non-coding transcriptome has been largely unexplored. Here, we demonstrate that bufalin suppresses ESCC progression by targeting the E2F2/LINC01410/β-catenin axis. Our study reveals three key mechanisms: (i) Bufalin downregulates the oncogenic lncRNA LINC01410; (ii) LINC01410 physically associates with and stabilises β-catenin, thereby activating Wnt signalling; and (iii) Bufalin represses LINC01410 transcription by inducing proteasomal degradation of its upstream transcriptional regulator, E2F2 (Fig. 8). Together, these findings provide a mechanistic rationale for considering bufalin as a targeted therapeutic strategy for ESCC.

RNA sequencing analysis identified LINC01410 as a primary downstream target of bufalin. Located on chromosome 1q25.2, LINC01410 functions as an oncogene in multiple tumour contexts^33,34^. For example, it promotes cervical cancer metastasis by sponging miR-532-5p to upregulate FASN^35^, modulates ErbB^36^, PTEN/AKT^37^, and Notch signalling in glioma and osteosarcoma. In ESCC, fibroblast-derived exosomal LINC01410 has been reported to enhance PKM2 expression and promote metastasis^16^. However, the intrinsic regulatory mechanisms governing LINC01410 within ESCC cells remained unclear.

We observed significant enrichment of the Wnt/β-catenin pathway in ESCC with high LINC01410 expression. To our knowledge, this study demonstrates that LINC01410 directly binds to β-catenin and enhances its protein stability, thereby activating Wnt/β-catenin signalling and promoting ESCC progression. The Wnt/β-catenin pathway is a well-established regulator of cell development, differentiation, and apoptosis, and plays a critical role in the initiation and progression of multiple malignancies, including ESCC^6,38–40^. Under canonical conditions, Wnt ligand binding to Frizzled and LRP5/6 receptors triggers Dishevelled (DVL) activation and recruitment of the β-catenin destruction complex—comprising Axin, GSK-3β, CK1, and APC—to the receptor complex. This process sequesters GSK-3 β, prevents β-catenin phosphorylation and degradation, and leads to cytoplasmic accumulation and subsequent nuclear translocation of β-catenin. In the nucleus, β-catenin associates with TCF/LEF transcription factors and co-activators such as Pygopus (Pygo) and Bcl-9 to drive the expression of downstream target genes, including c-Myc, Cyclin D1, and Slug^41–44^.

In contrast to this canonical upstream activation, our findings suggest that LINC01410 promotes Wnt pathway activation by acting downstream of receptor engagement. We show that LINC01410 directly interacts with cytosolic β-catenin, inhibits its K48-linked ubiquitination, and protects it from proteasome-mediated degradation. This stabilisation extends the half-life of β-catenin and facilitates its nuclear translocation, thereby driving transcription of downstream targets such as c-Myc and Cyclin D1. Although multiple ubiquitin ligases and deubiquitinases, including TRIM25^45^, USP10^46^, USP8^47^ and NDR1/FBXO11^48^, have been implicated in β-catenin turnover, our study identifies LINC01410 as a previously unrecognised RNA scaffold that sustains aberrant β-catenin signalling in ESCC. While previous studies indicated that bufalin inhibits ESCC via PIAS3-mediated STAT3 suppression^30^, our data elucidate its additional role in regulating the Wnt/β-catenin pathway. Given the documented crosstalk between STAT3 and Wnt/β-catenin signalling^49,50^, bufalin’s dual effects may involve interconnected regulatory mechanisms. Notably, LINC01410 overexpression reversed bufalin’s inhibitory effects, confirming that the suppression of the LINC01410-mediated Wnt/β-catenin pathway constitutes a primary mechanism by which bufalin exerts its anti-tumour effects in ESCC.

Furthermore, we identify E2F2 as the transcriptional driver of LINC01410 and a direct molecular target of bufalin. E2F2 is a key regulator of cell cycle progression, differentiation, and angiogenesis, and has been linked to aggressive tumour behaviour and poor prognosis^51^. In recent years, increasing evidence indicates that E2F2 promotes tumour cell proliferation, angiogenesis, and invasiveness, thereby playing a key role in the tumorigenesis and development of various malignancies^29^. Despite its oncogenic role, few agents directly target E2F2. Targeted protein degradation (TPD) has emerged as a promising strategy to eliminate such proteins in recent years^52^. Consistent with previous reports demonstrating that Bufalin functions as a molecular glue to promote the formation of the E2F2-ZFP91 complex^28^, or to promote E3 ligase-mediated degradation of ERα^18^ and EGFR^53^, our study shows that bufalin induces ubiquitination and proteasomal degradation of E2F2 in ESCC cells. Depletion of E2F2 leads to transcriptional silencing of LINC01410, thereby functionally suppressing the LINC01410/β-catenin signalling cascade.

In summary, this study demonstrates a previously unrecognised pharmacological pathway in which bufalin suppresses ESCC progression by targeting the E2F2/LINC01410/β-catenin axis. By inducing E2F2 degradation, bufalin transcriptionally downregulates LINC01410, destabilises β-catenin, and attenuates Wnt signalling. These findings provide strong mechanistic support for further clinical evaluation of bufalin as a Wnt pathway modulator in ESCC.

## Methods

### Reagents, antibodies, and cells

Bufalin (T1719) and the Wnt pathway agonist SKL2001 (T6989) were obtained from TargetMol (Boston, MA, USA). Antibodies used included: E-Cadherin (24E10, 3195S, 1:1000 for WB), N-cadherin (D4RIH, 13116S, 1:1000 for WB, 1:500 for IF), Slug (9585 T, 1:1000 for WB, 1:500 for IF) from CST (Danvers, MA, USA); CyclinD1 (60186-1-Ig, 1:5000 for WB), PCNA (60097-1-Ig, 1:10,000 for WB), c-MYC (10828-1-AP, 1:5000 for WB), Vimentin (60330-1-Ig, 1:20,000 for WB), CDK4 (66950-1-Ig, 1:5000 for WB), β-catenin (51067-2-AP, 1:5000 for WB, 1:1000 for IHC, 1:500 for IF), E-cadherin (60902-1-Ig, 1:10,000 for WB, 1:2000 for IF), IgG (30,000-0-AP) from Proteintech (Rosemont, IL, USA); Ki67 (ab16667, 1:200 for IHC), Goat Anti-Rabbit IgG H&L (Alexa Fluor® 488, ab150077, 1:200 for IF), Goat Anti-Mouse IgG H&L (Alexa Fluor® 647, ab150115, 1:200 for IF), Goat Anti-Rabbit IgG H&L (Alexa Fluor® 647, ab150079, 1:200 for IF), Goat Anti-Mouse IgG H&L (Alexa Fluor® 488, ab150113, 1:200 for IF) from abcam (Cambridge, MA, USA); GAPDH (D110016, 1:10,000 for WB) from Sangon Biotech (Zhengzhou, China); E2F2 (A3297, 1:1000 for WB) from Abdonal (Shanghai, China). Esophageal squamous cell carcinoma (ESCC) cell lines (EC-109, EC-9706) and human embryonic kidney cells (293 T) were purchased from the National Collection of Authenticated Cell Cultures, Chinese Academy of Sciences.

### Cell Culture

EC-109 and EC-9706 cells were maintained in RPMI-1640 medium (ZQ-200, Zhongqiao Xinzhou, Shanghai, China), and 293 T cells in DMEM high-glucose medium (ZQ-100, Zhongqiao Xinzhou, Shanghai, China). Both media were supplemented with 10% foetal bovine serum (RYS-KF21-01, Royacel, Lanzhou, China), 100 U/mL penicillin, and 100 μg/mL streptomycin (C0222, Beyotime, Shanghai, China). Cells were cultured at 37 °C in a 5% CO₂ incubator (3111, Thermo Fisher Scientific, USA). Cell morphology was monitored regularly under the microscope (Ts2, Nikon, Tokyo, Japan) to ensure exponential growth prior to experiments.

### Cell proliferation assay

EC-109 and EC-9706 cells in the exponential phase were seeded into 96-well plates (5 × 10³ cells/well). After 24 h, cells were treated with bufalin (0, 5, 10, 20, 50, 100 nM) for another 48 h. Proliferation was quantified using CCK-8 (MC0301-500, Kermy, Zhengzhou, China) and EdU detection kits (C0075L, Beyotime, Shanghai, China). For CCK-8, reagent (10 μL) was added for 2 h, and absorbance measured at 450 nm (Epoch2, Bio-Tek, Winooski, VT, USA). In the EdU, working solution was added and incubated at 37 °C for 2 h; then the cells were fixed with 4% paraformaldehyde for 15 min and permeabilized with 0.3% Triton X-100 for 15 min; 50 μL of Click reaction solution was added and incubated in the dark for 30 min, followed by Hoechst 33342 staining for 10 min in the dark; finally, the fluorescence signal was observed and recorded using an inverted fluorescence microscope (XDS-200C, Zeiss, Germany)^54^.

### Cell migration and invasion assays

The experiments were performed using Transwell cell culture inserts (3422, Corning Life Science, Corning, NY, USA). For the invasion assay, 50 μL of Matrigel (354234, BD Biosciences, USA) was added to the upper chamber and incubated at 37 °C for 30 min to allow it to solidify; the migration assay did not involve Matrigel coating. Cells treated with bufalin were diluted to 1 × 10⁵ cells/mL in serum-free medium, and 200 μL of the cell suspension was added to the upper chamber. 500 μL of complete culture medium containing bufalin was added to the lower chamber, and the cells were incubated in a cell culture incubator for 24 h. After incubation, non-migrated cells in the upper chamber were removed by wiping, and the cells on the lower surface of the membrane were fixed and stained with crystal violet. Images were captured under a microscope (Ts2, Nikon, Tokyo, Japan), and the cells were quantified using ImageJ software.

### RNA sequencing and sequence analysis

Total RNA was extracted from bufalin-treated EC-109 cells using TRIzol (9109, Takara, Dalian, China). RNA sequencing was conducted on the Illumina NovaSeq™6000 platform (LC-Bio Technology Co., Hangzhou, China). Differential expression was analysed using R software ( | fold change | ≤0.5, *P* < 0.05). LINC01410 levels in ESCC tissues were assessed via the Gene Expression Profiling Interactive Analysis (GEPIA) database (http://gepia.cancer-pku.cn/). Functional enrichment was performed using Gene Set Enrichment Analysis (GSEA) (http://www.broadinstitute.org/gsea/) with the DEG and ESCA cohorts from the TCGA dataset.

### Lentiviral transduction

Human LINC01410-targeting shRNA, negative control shRNA (shNC), and LINC01410 overexpression vectors (HBLV-ZsGreen-PURO) were constructed by Hanbio (Shanghai, China). Transduction was performed using polybrene, and stable lines were selected via puromycin. Efficiency was validated by qRT-PCR.

### qRT-PCR

Total RNA was extracted using TRIzol reagent (9109, Takara, Dalian, China), and reverse transcription was then performed with the kit (E047-01B, Novoprotein, Shanghai, China). Expression of LINC01410, GAPDH, and Wnt targets (Cyclin D1, c-Myc, Slug, MMP7) was quantified using SYBR qPCR SuperMix Plus (E096, Novoprotein, Shanghai, China). Primers were synthesised by Sangon Biotech (Zhengzhou, China), with the sequences as follows: LINC01410 (forward: 5’ → 3’ ACAGCCCTGAGTGAAAAGTCC, reverse: 5’ → 3’ ACAAGATGCCTTGGAGTGTCA), c-Myc (forward: 5’ → 3’ GGGCTTCTCAGAGGCTTGG, reverse: 5’ → 3’ GTCCTTGCTCGGGTGTTGTA), Cyclin D1 (forward: 5’ → 3’ AGAGGCGGAGGAGAACAAAC, reverse: 5’ → 3’ GGCGGATTGGAAATGAACTT), Slug (forward: 5’ → 3’ ATCTGCGGCAAGGCGTTTTCCA, reverse: 5’ → 3’ GAGCCCTCAGATTTGACCTGTC), MMP7 (forward: 5’ → 3’ TCGGAGGAGATGCTCACTTCGA, reverse: 5’ → 3’ GGATCAGAGGAATGTCCCATACC), U2 snRNA (forward: 5’ → 3’ TTGGAGCAGGGAGATGGAATAGGAG, reverse: 5’ → 3’ TGCACCGTTCCTGGAGGTACTG), GAPDH (forward: 5’ → 3’ GCACCGTCAAGGCTGAGAAC, reverse: 5’ → 3’ GCCTTCTCCATGGTGGTGAA).

### Immunofluorescence

Cells were fixed with 4% paraformaldehyde, permeabilized with 0.2% Triton X-100, and incubated overnight with primary antibodies at 4 °C. The next day, Alexa Fluor secondary antibodies (Abcam, Cambridge, MA, USA) were added for 1 h. Cell nuclei were counterstained with DAPI. Images were acquired using fluorescence and confocal laser scanning microscopes (XDS-200C, Zeiss, Germany; AX, Nikon, Japan).

### Western blot analysis

Cell lysates were prepared in RIPA buffer and protein concentrations determined using BCA protein assay kit (ZJ102, EpiZyme, Shanghai, China). Proteins were separated by SDS-PAGE, then transferred to PVDF membranes (IPVH00010, Millipore, Bedford, MA, USA), blocked, and incubated with antibodies. Protein expression bands were visualised using a chemiluminescence system (GE Amersham Imager 600, GE Healthcare, Chicago, USA).

### Fluorescence in situ hybridisation (FISH) test and subcellular fractionation

FISH detection was performed using a Cy3-labelled LINC01410 probe (GenePharma, Suzhou, China). Subcellular RNA was isolated using nuclear RNA purification kit (NGB-21000, Norgen Biotek, Thorold, ON, Canada), and the distribution of LINC01410 in the cytoplasm and nucleus was quantified by RT-qPCR.

### Chromatin immunoprecipitation (ChIP)

ChIP was conducted using a commercial kit (P2078, Beyotime, Shanghai, China). EC-109 cells were cross-linked with formaldehyde for 10 min, followed by the addition of glycine to stop the cross-linking. The cells were then sonicated to fragment the chromatin (100–500 bp). 1 mg of cell lysate was incubated with either E2F2 antibody (1.0 μg) or IgG control antibody (1.0 μg) overnight at 4 °C. Protein A/G agarose beads were added to capture the antibody-protein-DNA complexes. After washing, elution, and de-crosslinking, the DNA was extracted and analysed by qRT-PCR to determine the binding of E2F2 to the LINC01410 promoter.

### RNA immunoprecipitation (RIP)

RIP was performed according to the instructions of the BersinBio RIP kit (Bes5101, BersinBio, Guangzhou, China). Cells were lysed in lysis buffer containing protease and RNase inhibitors. After DNase digestion, 3 mg of cell lysate was incubated overnight at 4 °C with either a β-catenin antibody (4.0 μg) or an IgG control antibody (4.0 μg). Protein A/G agarose beads were then added to capture the complex. After washing, RNA was purified using an RNA extraction reagent, and the enrichment level of LINC01410 was assessed by qRT-PCR.

### RNA pull-down

To examine the interaction between LINC01410 and β-catenin, RNA Pull-down kit were used (Bes5102, BersinBio, Guangzhou, China). Briefly, biotin-labelled full-length LINC01410 RNA probes (sense) and their antisense RNA (negative control) were synthesised in vitro. The probes were then bound to streptavidin magnetic beads and incubated with total protein extracts from EC-109 cells to capture proteins bound to the RNA. The eluted products were first verified by silver staining, followed by Western blot analysis to determine the enrichment of β-catenin.

### Dual luciferase reporter assay

TCF/LEF-Luc reporter plasmid, LINC01410 wild-type/mutant plasmids, and Renilla luciferase internal control construct were mixed in a ratio of 25:25:1 and co-transfected into cells using Lip2000 transfection reagent (ML0201, Kermy, Zhengzhou, China). After 48 h of transfection, luciferase activity was measured using a dual-luciferase reporter gene assay kit (RG027, Beyotime, Shanghai, China). Firefly luciferase activity was normalised to Renilla luciferase activity to calculate the relative fluorescence intensity.

### Machine learning-based modelling, interpretation, and feature importance analysis

This study was conducted in the R programming environment and trained on a merged transcriptomic dataset, primarily using TCGA-ESCA esophageal cancer RNA-seq data (https://portal.gdc.cancer.gov/projects/TCGA-ESCA) as the main training cohort. The ‘train‘ function from the ‘caret‘ package was used for unified training and performance evaluation of various machine learning models, including ten common classification models: Generalised Linear Model (GLM), Logistic Regression (Logit), Random Forest (RF), Gradient Boosting Machine (GBM), Support Vector Machine (SVM), K-Nearest Neighbours (KNN), Elastic Net Regression, Naive Bayes, Partial Least Squares (PLS), and stepwise Linear Discriminant Analysis (stepLDA). Consistent feature input matrices and response variables were used to ensure comparability between different models. During model training, a 10-fold cross-validation strategy was employed to optimise hyperparameters, improving the model’s generalisation ability and reducing the risk of overfitting. Necessary standardisation of continuous variables was performed according to the characteristics of different algorithms.

For model interpretation, the ‘explain‘ function from the DALEX package was used to uniformly interpret the various models trained under the caret framework, extracting stable and comparable feature contribution information from the model’s interpretability. Model prediction performance was evaluated using the built-in ‘predict‘ function in R, and the receiver operating characteristic (ROC) curve was used to analyse the model’s discriminative ability, calculating the area under the curve (AUC) to quantify the classification performance of each model.

To comprehensively assess the model error structure and stability, we further plotted the reverse cumulative distribution of residuals and residual box plots to visually compare the performance of different models in terms of overall error level and coverage of low-error samples.

Feature importance analysis was performed using the ‘variable_importance‘ function in the DALEX package. This method is based on the permutation importance principle, assessing the impact of a single variable’s absence on the prediction results of the response variable by randomly shuffling that variable and recalculating the model prediction error. This study used the root mean square error (RMSE) as the loss function; the increase in RMSE reflects the degree of contribution of that variable to the model’s predictive performance. Specifically, if the model’s RMSE significantly increases after shuffling a variable, it indicates that the variable has high importance in the model. Based on the methods described above, we calculated and outputted the ranking results of important features (genes) in each machine learning model. By integrating the consistency of feature importance across multiple models, we selected important candidate genes that performed stably under different algorithmic frameworks, thereby improving the robustness and biological credibility of the results.

### In vivo tumour growth and metastatic potential assays

All animal experiments in this study strictly followed the *Health Guide for the Care and Use of Laboratory Animals* and the ARRIVE guidelines (https://arriveguidelines.org), and were approved by the Institutional Animal Care and Use Committee of The First Affiliated Hospital of Xinxiang Medical University (Approval No.: EC-2021-706).

Specific pathogen-free (SPF) male BALB/c nude mice (4–6 weeks old) were purchased from Beijing Vital River Laboratory Animal Technology Co., Ltd. All mice were housed in the experimental animal centre of The First Affiliated Hospital of Xinxiang Medical University under constant environmental conditions: temperature (24 ± 2) °C, 12-h light/dark cycle. Mice had free access to standard irradiated feed and sterile water. To evaluate the inhibitory effect of bufalin on tumour growth, EC-109 cells in the logarithmic growth phase were resuspended in a mixture of serum-free RPMI-1640 medium and Matrigel matrix gel (1:1 volume ratio). After grouping the mice using a random number table, a suspension containing 5 × 10⁶ cells (total volume 100 μL) was subcutaneously injected into the right flank of each mouse. The health status of the mice was observed daily after inoculation. When the average tumour volume (volume = length × width²/2) reached approximately 100 mm³, the mice were randomly divided into two groups (*n* = 6 mice/group): control group (intraperitoneal injection of physiological saline) and bufalin treatment group (1.0 mg/kg/day, intraperitoneally)^21^. The day of grouping was recorded as treatment day 0. Tumour volume and mouse weight were measured twice a week to assess tumour growth and potential drug toxicity. The treatment period lasted for 4 weeks. To evaluate the inhibitory effect of bufalin on distant tumour metastasis, another batch of mice was used, and 2 × 10⁶ EC-109 cells were injected via the tail vein. Twenty-four hours after cell injection, the mice were randomly divided into a control group and a bufalin treatment group (grouping method, dosage, and route of administration were the same as in the subcutaneous xenograft model). The treatment period lasted for 6 weeks. After the treatment, all mice were euthanised by carbon dioxide inhalation^55^. Subsequently, the subcutaneous xenografts were completely dissected and weighed; lung tissues were also collected. Some tumour tissue samples were snap-frozen in liquid nitrogen and then stored at −80 °C for subsequent molecular biological analysis; other tumour tissue samples and all lung tissue samples were fixed in 4% paraformaldehyde solution for more than 24 h, followed by paraffin embedding for histological analysis (hematoxylin-eosin staining and immunohistochemical staining). Lung metastases were evaluated using H&E-stained sections.

### Immunohistochemical (IHC) staining

Tumour tissue was fixed, paraffin-embedded, and sectioned. After deparaffinisation and rehydration, antigen retrieval was performed, followed by incubation with the primary antibody. Biotin-labelled anti-rabbit IgG secondary antibody was added, and visualisation was performed using a DAB chromogenic kit. IHC results were scored according to previously established criteria^56^.

### Statistical analysis

Data are presented as mean ± standard deviation (SD). For comparisons between two independent conditions or groups, a two-tailed unpaired Student’s t-test was applied. For analyses involving more than two groups, one-way or two-way analysis of variance (ANOVA) was performed, followed by Dunnett’s multiple comparison test to determine specific group differences. All statistical analyses were performed using GraphPad Prism version 8.0 (GraphPad Software, San Diego, USA). A p-value < 0.05 was considered statistically significant, indicated as *p < 0.05, **p < 0.01, and ***p < 0.001.

## Supplementary information

Supplementary.

Supplementary Table S2

## Data Availability

The datasets used in this paper are available online, as described in the “Methods” section. No new algorithms were developed for this article. All code generated for analysis is available from the corresponding author upon request.

## Code availability

No new algorithms were developed for this article. All code generated for analysis is available from the corresponding author upon request.
