# Supplementary material for: Bufalin targets E2F2 to transcriptionally inhibit LINC01410 and suppress Wnt/β-catenin signaling in esophageal squamous cell carcinoma
Source: NPJ Precis Oncol. 2026 May 18;10:303. doi: 10.1038/s41698-026-01395-0 (PMC13448694; doi:10.1038/s41698-026-01395-0)
Supplement: Supplementary file 1 [file 41698_2026_1395_MOESM1_ESM.docx]

**Bufalin targets E2F2 to transcriptionally inhibit LINC01410 and suppress Wnt/β-catenin signaling in esophageal squamous cell carcinoma**

# Supplementary Figure S1 | Bufalin inhibits the proliferation, migration and invasion of EC-9706 cells. (A) CCK-8 assay after 48 h of treatment with various concentrations of bufalin; (B) EdU (red) and Hoechst (blue) staining of EC-9706 cells treated with bufalin; (C) Cell cycle histography analysis; (D) Protein levels of Cyclin D1, CDK4, and PCNA in EC-9706 cells after bufalin treatment, detected by Western blot (GAPDH used as internal control); (E) Migration and invasion abilities of EC-9706 cells after bufalin treatment, detected by Transwell assay; (F) Protein expression levels of E-cadherin, N-cadherin, and Vimentin analyzed by Western blot; (G) Immunofluorescence staining of E-cadherin (red), N-cadherin (red), and DAPI (blue) in bufalin-treated cells.

# Supplementary figure S2 | Signaling pathway enrichment. Using GSEA, the NES enrichment score was used to rank the WNT signaling pathway first in patients with high linc01410 expression.

# Supplementary figure S3 | LINC01410 co-localizes with β-catenin. LINC0140 localization was first detected by a FISH kit using probes for cyc3-labeled LINC0140 in EC-109 cells, followed by β-catenin localization by immunofluorescence.

# Supplementary figure S4 | Comparison of residual distribution and prediction accuracy among 10 machine learning models.(A) Box plot of absolute residuals (|residual|) of the 10 machine learning models. The box plot shows the distribution of absolute residuals of the 10 models, and red dots represent the root mean square error (RMSE) of residuals; (B) Reverse cumulative distribution curves of residuals of the 10 machine learning models. Reverse cumulative distribution curves of residuals (|residual|) were used to compare the prediction accuracy of different models.

# Supplementary figure S5 | Expression levels of E2F2 and LINC01410 in different clinical stages of esophageal cancer. (A) Expression levels of LINC01410 in normal esophageal tissues and esophageal cancer tissues at different clinical stages (Stage1-Stage4) from the TCGA cohort (expressed as FPKM values); sample sizes of each group: Normal (n=11), Stage1 (n=16), Stage2 (n=69), Stage3 (n=49), Stage4 (n=8). Statistical analysis showed significant differences in LINC01410 expression between Normal-vs-Stage1 (p=0.02316), Normal-vs-Stage2 (p=0.00001), Normal-vs-Stage3 (p=0.00002), and Normal-vs-Stage4 (p=0.01307); (B) Expression levels of E2F2 in normal esophageal tissues and esophageal cancer tissues at different clinical stages from the TCGA cohort (expressed as Transcript per million values); sample sizes of each group: Normal (n=11), Stage1 (n=13), Stage2 (n=78), Stage3 (n=55), Stage4 (n=9). Statistical analysis showed significant differences in E2F2 expression between Normal vs Stage1 (p=0.00126), Normal vs Stage2 (p=0.00002), Normal vs Stage3 (p=0.00314), and Normal vs Stage4 (p=0.00076).

# Supplementary figure S6 | Bufalin degrades E2F2 via the ubiquitin lysosomal pathway. (A) Cells were incubated with bufalin in combination with the proteasome inhibitor MG132, and lysates were analyzed for E2F2 expression; (B) Cells were incubated with bufalin combined with the proteasome inhibitor chloroquine, and the lysates were analyzed for E2F2 expression.

# Supplementary Table S1.

Molecular docking study

| Protein ID | Ligand | | Binding Affinity | rmsd/ub | rmsd/lb |
| --- | --- | --- | --- | --- | --- |
| E2F2 | 1n4m2_9547215_uff_E=664.02_uff_E=663.93 | -8.6 | | 0 | 0 |
|  | 1n4m2_9547215_uff_E=664.02_uff_E=663.93 | -8.4 | | 7.731 | 4.132 |
|  | 1n4m2_9547215_uff_E=664.02_uff_E=663.93 | -8.4 | | 3.718 | 2.093 |
|  | 1n4m2_9547215_uff_E=664.02_uff_E=663.93 | -8.4 | | 8.198 | 4.13 |
|  | 1n4m2_9547215_uff_E=664.02_uff_E=663.93 | -8.4 | | 10.698 | 8.209 |
|  | 1n4m2_9547215_uff_E=664.02_uff_E=663.93 | -8.2 | | 6.078 | 3.827 |
|  | 1n4m2_9547215_uff_E=664.02_uff_E=663.93 | -8.1 | | 16.038 | 11.781 |
|  | 1n4m2_9547215_uff_E=664.02_uff_E=663.93 | -8.1 | | 9.07 | 6.645 |
|  | 1n4m2_9547215_uff_E=664.02_uff_E=663.93 | -8 | | 6.567 | 3.863 |
| IRF1 | IRF1F_9547215_uff_E=664.02_uff_E=663.93 | -7.9 | | 0 | 0 |
|  | IRF1F_9547215_uff_E=664.02_uff_E=663.93 | -7.8 | | 20.813 | 17.387 |
|  | IRF1F_9547215_uff_E=664.02_uff_E=663.93 | -6.9 | | 4.284 | 2.639 |
|  | IRF1F_9547215_uff_E=664.02_uff_E=663.93 | -6.8 | | 8.628 | 2.818 |
|  | IRF1F_9547215_uff_E=664.02_uff_E=663.93 | -6.7 | | 28.214 | 25.346 |
|  | IRF1F_9547215_uff_E=664.02_uff_E=663.93 | -6.6 | | 7.252 | 3.211 |
|  | IRF1F_9547215_uff_E=664.02_uff_E=663.93 | -6.6 | | 19.816 | 17.382 |
|  | IRF1F_9547215_uff_E=664.02_uff_E=663.93 | -6.6 | | 19.017 | 16.125 |
|  | IRF1F_9547215_uff_E=664.02_uff_E=663.93 | -6.5 | | 16.835 | 13.868 |
| HNF1B | HNF1B_model1_9547215_uff_E=664.02_uff_E=663.93 | -7.7 | | 0 | 0 |
|  | HNF1B_model1_9547215_uff_E=664.02_uff_E=663.93 | -7.6 | | 17.528 | 13.787 |
|  | HNF1B_model1_9547215_uff_E=664.02_uff_E=663.93 | -7.4 | | 17.265 | 13.998 |
|  | HNF1B_model1_9547215_uff_E=664.02_uff_E=663.93 | -7.3 | | 15.833 | 13.24 |
|  | HNF1B_model1_9547215_uff_E=664.02_uff_E=663.93 | -7.2 | | 17.022 | 13.234 |
|  | HNF1B_model1_9547215_uff_E=664.02_uff_E=663.93 | -7 | | 15.013 | 12.86 |
|  | HNF1B_model1_9547215_uff_E=664.02_uff_E=663.93 | -7 | | 7.259 | 1.578 |
|  | HNF1B_model1_9547215_uff_E=664.02_uff_E=663.93 | -7 | | 7.43 | 2.219 |
|  | HNF1B_model1_9547215_uff_E=664.02_uff_E=663.93 | -6.7 | | 7.372 | 2.169 |

# Animal Experiment Ethical Statement

# Original Western Blots

Figure 1. (D) Protein levels of Cyclin D1, CDK4, and PCNA in EC-109 cells after bufalin treatment, detected by Western blot (GAPDH used as internal control); (F) Protein expression levels of E-cadherin, N-cadherin, and Vimentin analyzed by Western blot.

Supplementary Figure S1. Protein levels of Cyclin D1, CDK4, and PCNA in EC-9706 cells after bufalin treatment, detected by Western blot (GAPDH used as internal control); (F) Protein expression levels of E-cadherin, N-cadherin, and Vimentin analyzed by Western blot.

Figure 2. (G) Protein levels of Cyclin D1, CDK4, PCNA, N-cadherin, and Vimentin in LINC01410-overexpressing cells after bufalin treatment, detected by Western blot (GAPDH used as internal control).

Figure 3. (H-I) Protein levels of Wnt target genes in cells with LINC01410 overexpression or knockdown, detected by Western blot (GAPDH used as internal control).

Figure 4. (E) Silver staining of RNA pull-down products after PAGE electrophoresis, followed by Western blot detection of β-catenin; (F) Stability of β-catenin protein in LINC01410-overexpressing cells, evaluated by CHX chase assay; (G) Expression of β-catenin in LINC01410-knockdown cells co-incubated with the proteasome inhibitor MG132 or the lysosome inhibitor chloroquine, analyzed by Western blot; (H) Ubiquitination level of β-catenin in 293T cells with LINC01410 knockdown or overexpression, detected by co-immunoprecipitation assay; (J) Distribution of β-catenin in the cytoplasm and nucleus of EC-109 cells, analyzed by Western blot (GAPDH used as cytoplasmic internal control; Histone H3 used as nuclear internal control).

Figure 5. (H) Protein levels of Wnt targets in cells co-treated with SKL2001 and bufalin, detected by Western blot (GAPDH used as internal control).

Figure 7. (A) Protein level of E2F2 in cells treated with bufalin, detected by Western blot (GAPDH used as internal control); (B) Stability of E2F2 evaluated by CHX chase assay (Ctrl: control group; BF: bufalin-treated group).

**Note:** The images in each set were derived from the same membrane and assembled together, with the assembly boundaries marked by black lines.

# Supplementary figure S6 | Bufalin degrades E2F2 via the ubiquitin lysosomal pathway. (A) Cells were incubated with bufalin in combination with the proteasome inhibitor MG132, and lysates were analyzed for E2F2 expression; (B) Cells were incubated with bufalin combined with the proteasome inhibitor chloroquine, and the lysates were analyzed for E2F2 expression.

**Note:** The images in each set were derived from the same membrane and assembled together, with the assembly boundaries marked by black lines.
